# Supplementary material for: The New Aristocrat of Wuyi Rock Tea: Chemical Basis of the Unique Aroma Quality of “Laocong Shuixian”
Source: Foods. 2025 May 12;14(10):1706. doi: 10.3390/foods14101706 (PMC12111090; doi:10.3390/foods14101706)
Supplement: Supplementary file 1 [file foods-14-01706-s001.zip › Supplementary figures.pdf]

Figure S1: Sensory evaluation of 12 LCSX tea sample purchased from the tea market

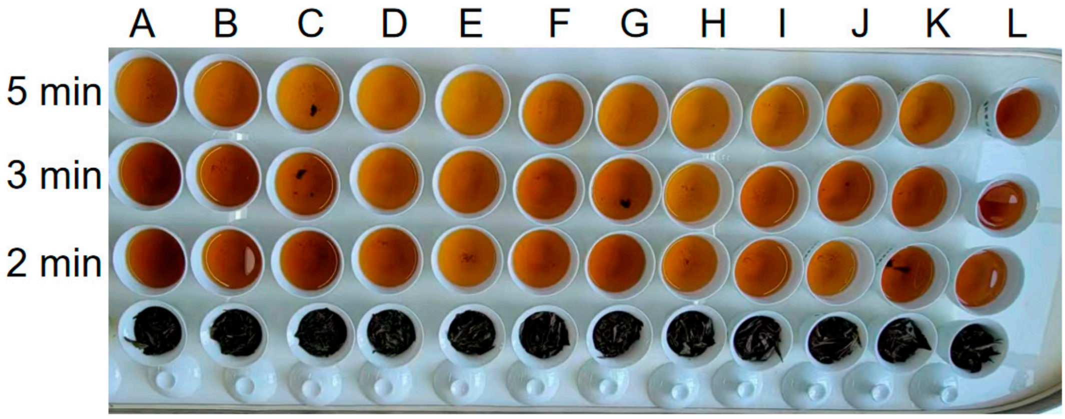

Figure S2: EI mass spectra of the most important compounds

Benzaldehyde RT: 23.84min

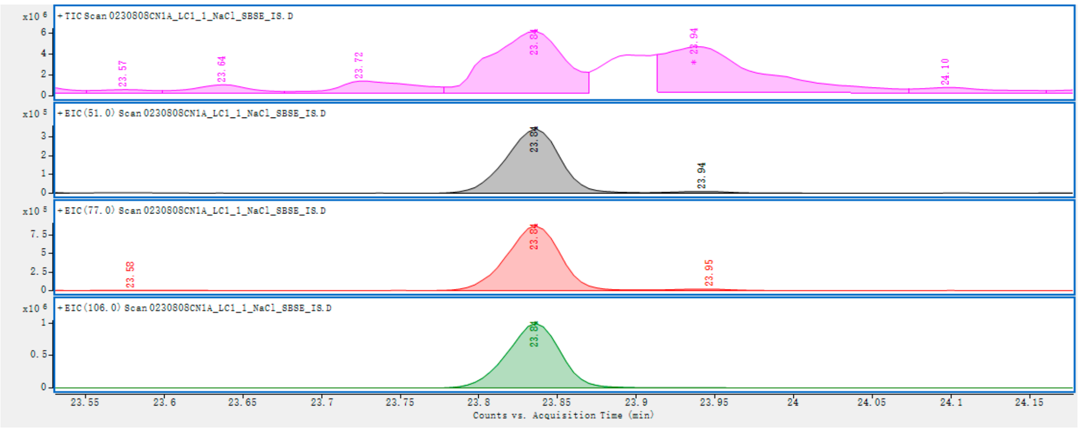

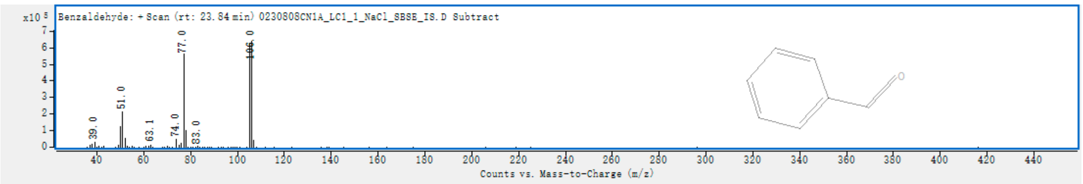

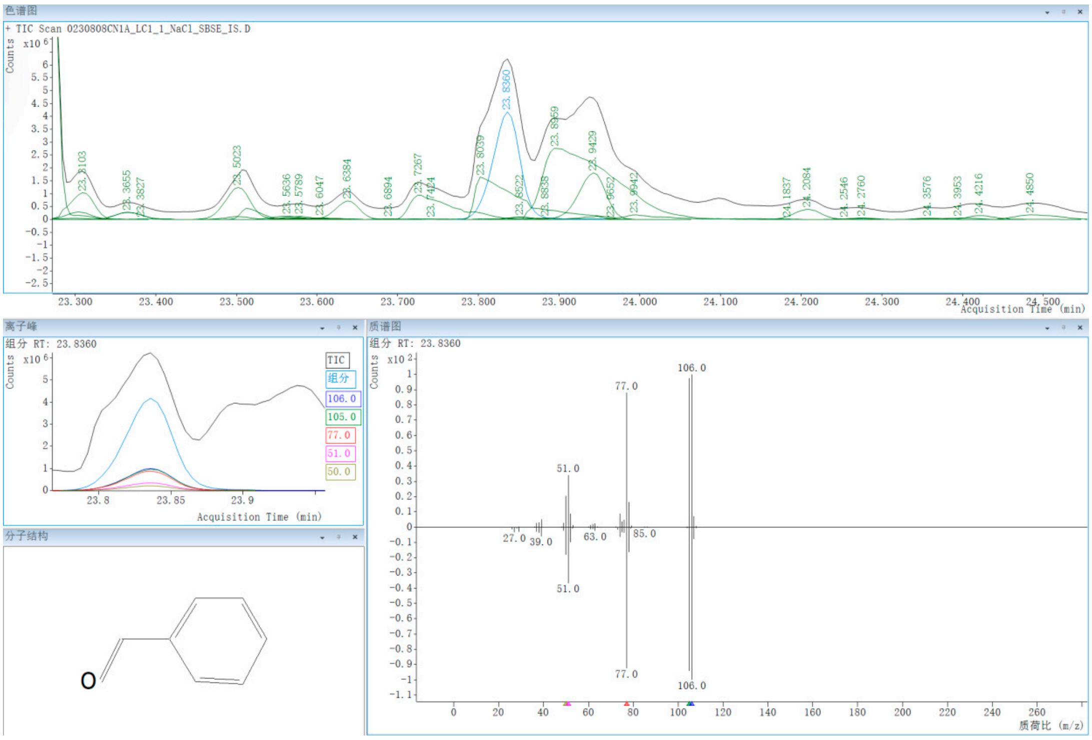

Geraniol RT: 30.8

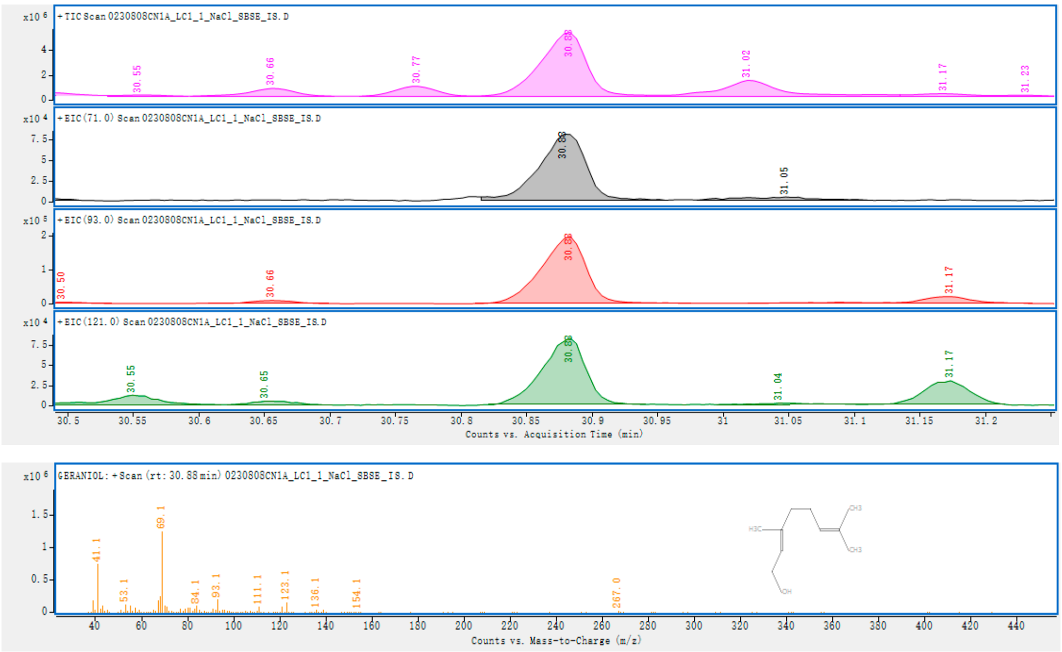

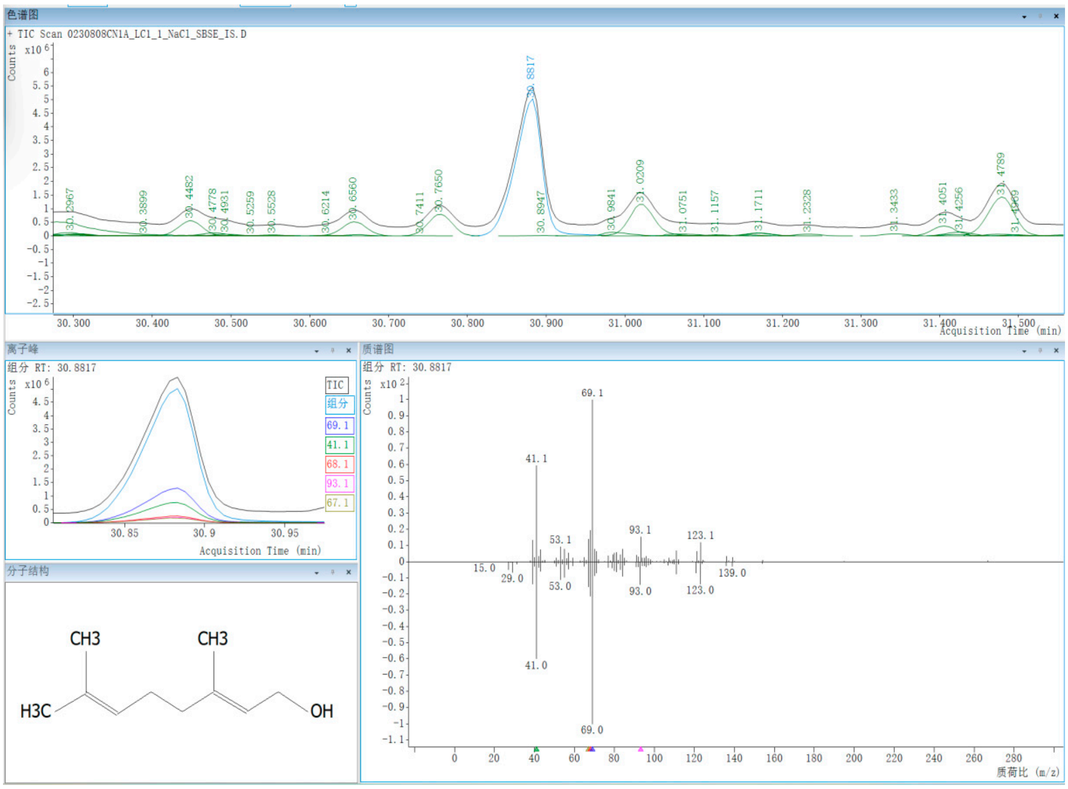

2-Acetylpyrrole RT:33.8

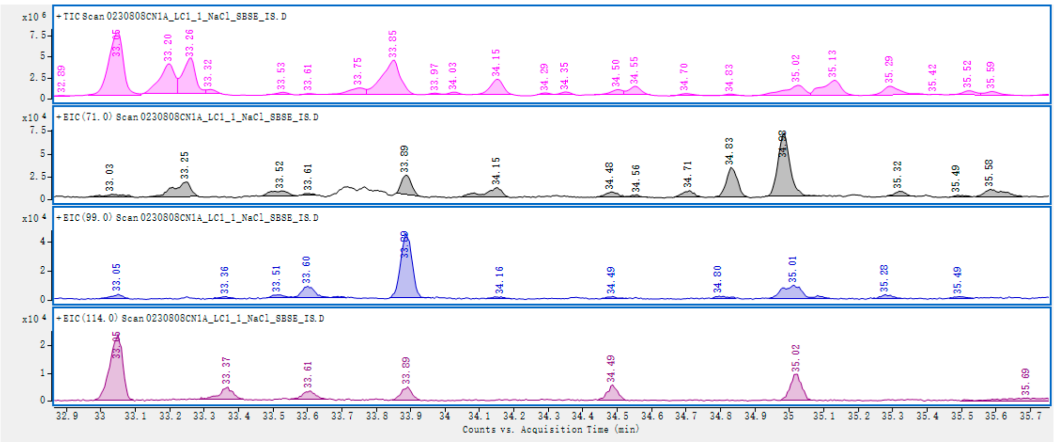

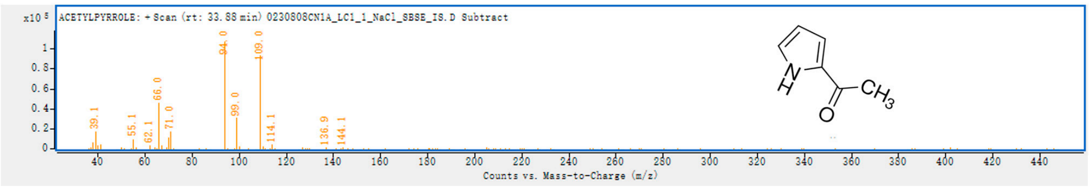

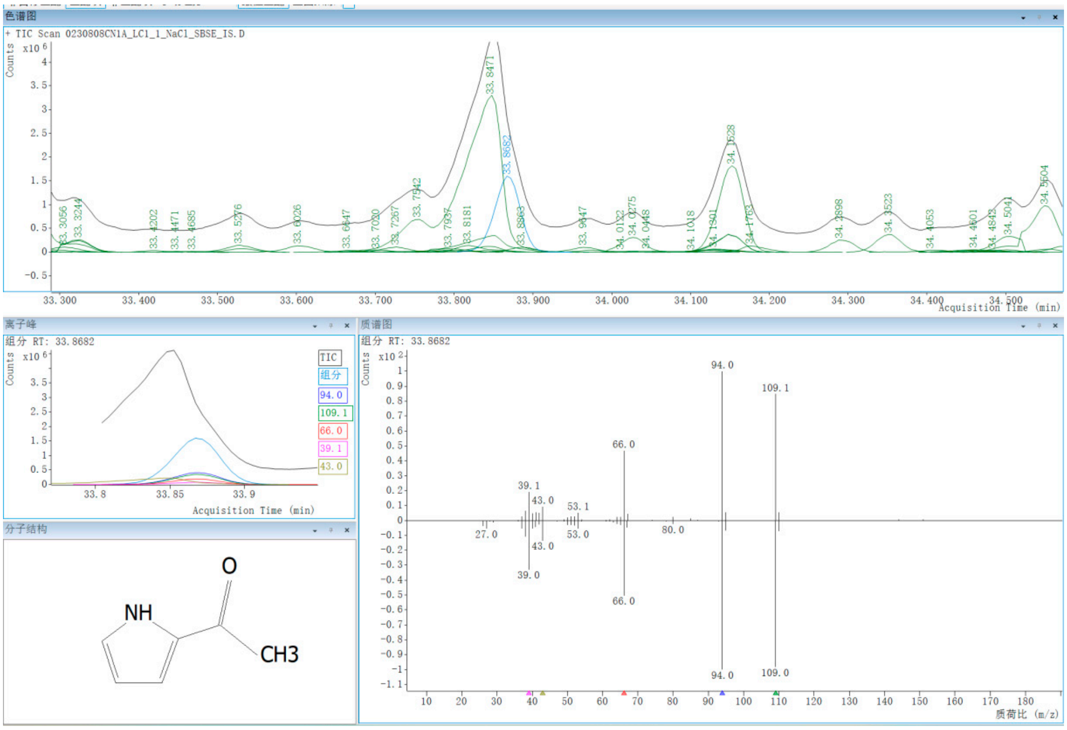

Theaspirone RT：37.99

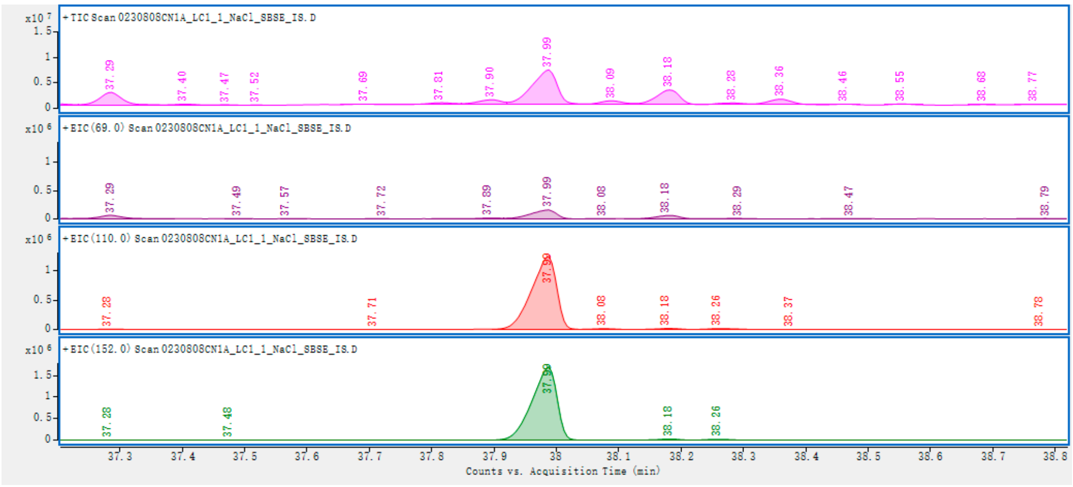

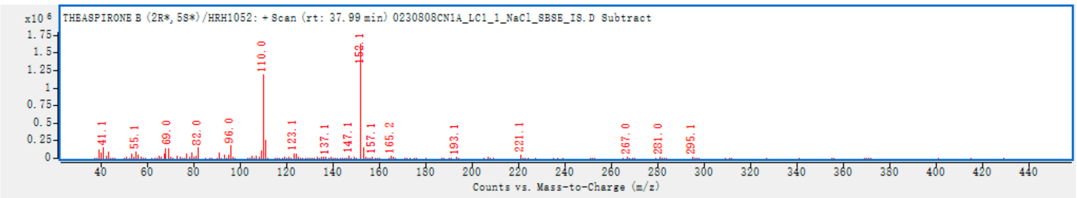
THEASPIRONE B (2R*, 5S*)/HRH1052: + Scan (rt: 37.99 min) 0230808CN1A_LC1_1_NaCl_SBSE_IS.D Subtract
Counts vs. Mass-to-Charge (m/z)

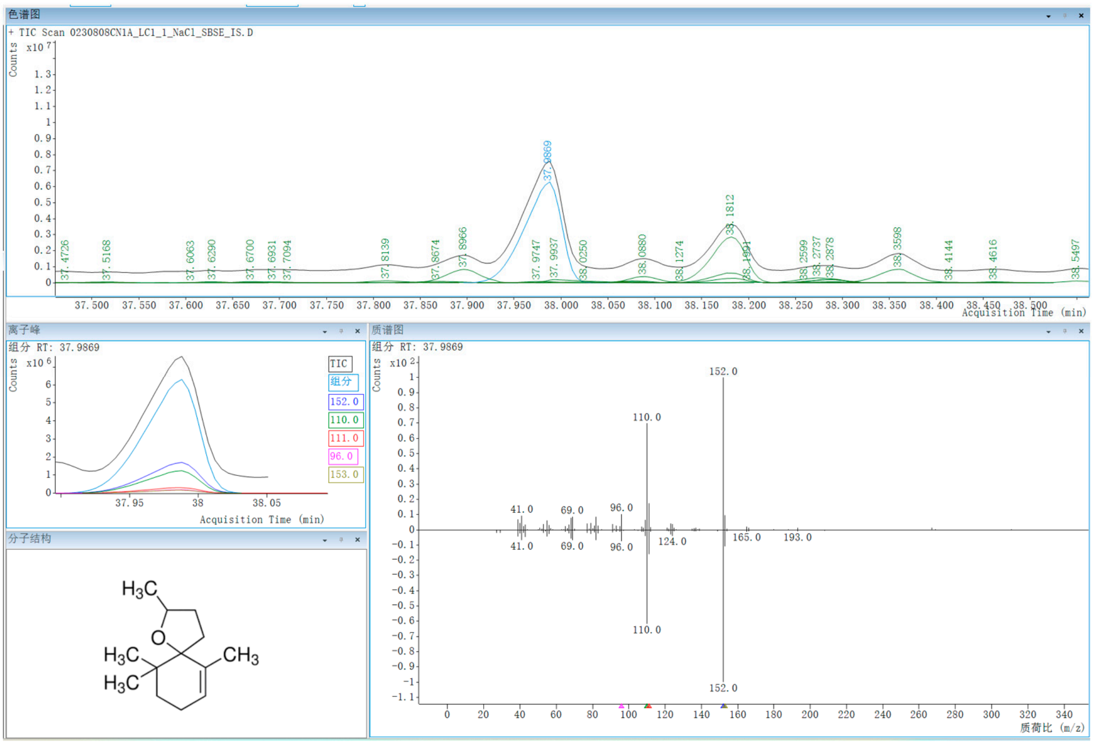
色谱图
+ TIC Scan 0230808CN1A_LC1_1_NaCl_SBSE_IS.D
Acquisition Time (min)
离子峰
组分 RT: 37.9869
TIC
组分
152.0
110.0
111.0
96.0
153.0
质谱图
组分 RT: 37.9869
质荷比 (m/z)
分子结构
